# Evaluation of the Modified Early Warning Score (MEWS) in In-Hospital Cardiac Arrest in a Tertiary Healthcare Facility

**DOI:** 10.3390/jcm14155384

**Published:** 2025-07-30

**Authors:** Osakpolor Ogbebor, Sitara Niranjan, Vikram Saini, Deeksha Ramanujam, Briana DiSilvio, Tariq Cheema

**Affiliations:** 1 Department of Pulmonary and Critical Care Medicine, Allegheny Health Network, Pittsburgh, PA 15212, USA; 2 Department of Internal Medicine, Allegheny Health Network, Pittsburgh, PA 15212, USA

**Keywords:** modified early warning score, early warning score, cardiac arrest, remote monitoring, critical care

## Abstract

**Background/Objective**: In-hospital cardiac arrest has high incidence and poor survival rates, posing a significant healthcare challenge. It is important to intervene in the hours before the cardiac arrest to prevent poor outcomes. The modified early warning score (MEWS) is a validated tool for identifying a deteriorating patient. It is an aggregate of vital signs and level of consciousness. We retrospectively evaluated MEWS for trends that might predict patient outcomes. **Methods**: We performed a single-center, one-year, retrospective study. A comprehensive review was conducted for patients aged 18 years and above who experienced a cardiac arrest. Cases that occurred within an intensive care unit, emergency department, during a procedure, or outside the hospital were excluded. A total of 87 cases met our predefined inclusion criteria. We collected data at 12 h, 6 h and 1 h time periods prior to the cardiac arrest. A trend analysis using a linear model with analysis of variance with Bonferroni correction was performed. **Results**: Out of 87 patients included in the study, 59 (67.8%) had an immediate return of spontaneous circulation (ROSC). Among those who achieved ROSC, 41 (69.5%) died during the admission. Only 20.7% of the patients that sustained a cardiac arrest survived to discharge. A significant increase in the average MEWS was noted from the 12 h period (MEWS = 3.95 ± 2.4) to the 1 h period (MEWS = 5.98 ± 3.5) (*p* ≤ 0.001) and the 6 h period (4.65 ± 2.6) to the 1 h period (5.98 ± 3.5) (*p* = 0.023) prior to cardiac arrest. **Conclusions**: An increase in the MEWS may be a valuable tool in identifying at-risk patients and provides an opportunity to intervene at least 6 h before a cardiac arrest event. Further research is needed to validate the results of our study.

## 1. Introduction

In the United States, 200,000 patients experience in-hospital cardiac arrests (IHCAs) annually, with a survival rate of 10–12% [1,2]. Among patients who have experienced an in-hospital cardiac arrest, there is significant clinical decline approximately 15 min prior to the cardiac arrest [3]. In such a short time frame, there is insufficient time for mitigating measures [3]. However, studies have demonstrated that patients may show signs of clinical deterioration hours prior to cardiac arrest, and these signs can be identified by systems that are triggered by alert parameters [4,5]. Many hospitals have introduced rapid response systems that use alert parameters to identify clinically deteriorating patients [6,7]. This can be based on a single parameter such as blood pressure and heart rate or a conglomerate of vital signs that culminate in an early warning score. Using an early warning score has the potential to identify deteriorating patients earlier compared to single vital parameters. Here, we evaluate the efficacy of an early warning score in predicting cardiac arrest over a 12 h period.

The modified early warning score (MEWS) is an aggregate of vital signs consisting of respiratory rate, oxygen saturation, systolic blood pressure, pulse rate, level of consciousness or new confusion, and temperature (Table 1) [8]. MEWS is a validated tool for identifying a deteriorating patient and facilitating early intervention prior to further clinical deterioration. A MEWS of 5 or more or a single physiological parameter score of 3 is associated with a higher relative risk. The aggregated score from the MEWS chart is intended to trigger appropriate management strategies and anticipate future resuscitation needs. Since MEWS describes clinical status, a static or deteriorated clinical status may warrant escalation to a higher level of care, with the hope of avoiding a negative outcome. MEWS provides a sensitive measure to detect subtle changes in a patient’s clinical condition that are likely to lead to hemodynamic or respiratory instability [9].

Using MEWS to assess cardiac-arrest risk on admission and for appropriate transfer is well described in the literature, but there are limited data on the how trending MEWS predict cardiac arrest and, specifically, the time period an intervention can mitigate a cardiac event. We hypothesized that a clinically deteriorating patient may have increasing MEWS. To retrospectively study trends, we analyzed and compared MEWS at 12, 6 and 1 h intervals prior to cardiac arrest. This study aims to examine the temporal variation in MEWS amongst patients who experience an in hospital cardiac arrest with the objective to identify potential threshold values that will trigger a clinical assessment and intervention. This study also explored the association of other factors such as comorbidities (diabetes, chronic kidney disease, severe acute respiratory syndrome coronavirus 2 (SARS-CoV-2) infection) and weight class that may affect outcomes.

## 2. Methods

We conducted a retrospective review of patients from 1 July 2020 to 30 June 2021, with a diagnosis of cardiac arrest within our hospital. Cardiac arrest International Classification of Diseases (ICD) 10 codes (I46, I46.9, I46.0, I46.1, I46.2, I46.8) were used to pool a population sample from our electronic medical record. All patients aged 18 years and above that had a cardiac arrest during the study period within our hospital system, had a systematic chart review. If the patient had multiple cardiac arrests, only the initial event was used in the analysis. The primary aim of the study was to identify patients that could deteriorate and progress to a cardiac arrest event and therefore implement measures to mitigate this outcome. An anticipated intervention would include escalation of care to the intensive care unit. Our hypothesis also focuses on predicting clinical deterioration prior to cardiac arrest occurring rather than responding to cases where the event is precipitated by identifiable provoking etiology such as complications from procedures. In view of this, patients were excluded from the study if the cardiac arrest occurred in an intensive care unit, emergency department, during a procedure, or outside the hospital. Cases from a different hospital were also excluded if vital signs documentation was not available.

The population of our study consisted of both surgical and medical patients. We collected patient demographic data, including age, race, sex, weight, comorbidities (hypertension, diabetes, and chronic kidney disease), and COVID-19 status. Weight was divided into four classes based on body mass index scores (BMI): underweight (<18.5), healthy weight (18.5–24.9), overweight (25–29.9), and obese (30 and above). To establish a trend in MEWS, vital signs (consciousness level, blood pressure, heart rate, respiratory rate, and temperature) were collected at 1, 6 and 12 h intervals before the cardiac arrest event (Table 1). Vitals were collected within 3 windows: 12 to 9 h, 6 to 3 h, and 2 h to 30 min before the event. These intervals represent MEWS at 12 h, 6 h, and 1 h periods, respectively. At least a 3 h time difference between each vital sign period block was maintained using the closest vital signs measured prior to that time point. To avoid inappropriate overestimation of MEWS, unavailable data were assumed to be normal for both mental status measurements (alert, verbal, pain, unconscious scoring or Glasgow coma scale scores) and temperature measurements.

### Statistical Analysis

This exploratory study employed a repeated measures design without a matched control group. Each case in the study population was evaluated at three distinct time periods, resulting in a total of 261 MEWS observations. Comparisons were conducted across all three time periods to assess temporal changes within the study population. Continuous variables were expressed as means with standard deviations. Comparisons of means were carried out using the independent samples *t*-test for variables consisting of two groups or analysis of variance (ANOVA) if a variable consisted of more than two groups. A trend analysis was conducted using a linear model with analysis of variance (ANOVA) with post hoc pairwise comparisons performed using the Bonferroni correction to adjust for multiple testing [10]. All tests of significance used a 2-sided *p* = 0.05. SPSS version 26 was used for all data analysis.

## 3. Results

**Figure 1:**
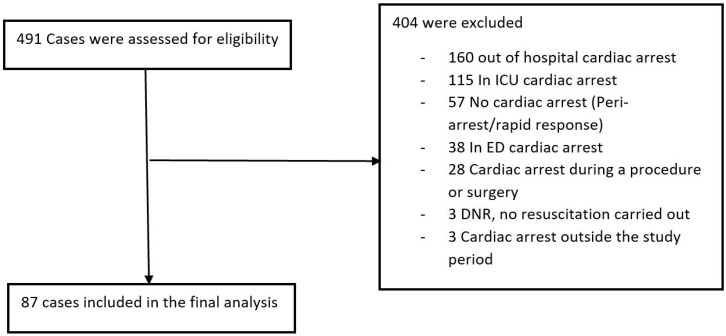
Screening for cardiac arrest cases for a retrospective chart review on the effectiveness of continuous modified early warning score monitoring in predicting cardiac arrest outcomes. (DNR: Do not resuscitate, ED: emergency department, ICU: intensive care unit).

In total, 491 patients had been assigned an ICD-10 code corresponding to cardiac arrest from 1 July 2020 through 30 June 2021. Of these, 404 patients were excluded based on location of the event. There were 87 cases of cardiac arrest that met our inclusion criteria (Figure 1).

Of these, 40.2% were female. Ages ranged from 19 to 102 years with a mean age of 69.3 ± 15 years. The average age at in-hospital cardiac arrest was 61.5 years for Black patients and 71.2 years for White patients (*p* = 0.016; Table 2). Regarding comorbidities, 9 patients had chronic kidney disease, of which 6 were on some form of renal replacement therapy prior to the cardiac arrest event. The median time to cardiac arrest was 5 days (range: 0 to 79 days) after admission to the hospital.

We evaluated the presenting rhythm, survival outcomes, and MEWS in our cohort. Most cases (*n* = 56, 64.4%) had a non-shockable rhythm, while 16 cases (18.4%) had a shockable rhythm. In 15 cases (17.2%) the rhythm was undocumented. Most patients (*n* = 59, 67.8%) had a return of spontaneous circulation (ROSC) as the immediate outcome of the resuscitation process, and these patients were transferred to an intensive care unit for a higher level of care. Amongst those in whom ROSC was achieved, 41 died during the same admission. Only 18 patients (20.7%) of the entire cohort survived and were discharged. The patients that survived to discharge had a lower MEWS compared to those who died during the admission. However, this finding was not statistically significant (Table 3).

**Figure 2:**
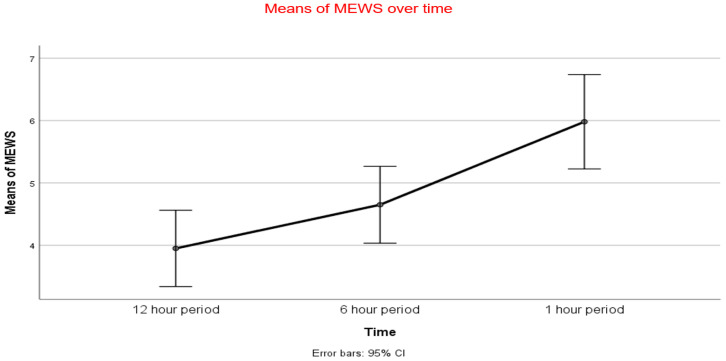
Trend of mean modified early warning score (MEWS) at 12 h, 6 h, and 1 h before cardiac arrest. MEWS significantly increased between the 12 h period/6 h period and the 1 h period, but not between the 12 h period and 6 h period.

When comparing MEWS during each interval, there was an overall significant rise in the average MEWS from the 12 h period (3.95 ± 2.4) to the 1 h period (5.98 ± 3.5) prior to cardiac arrest event, and from the 6 h period (4.65 ± 2.6) and 1 h period (5.98 ± 3.5; Figure 2).

Using Bonferroni correction, we identified a significant trend between the 12 h period and 1 h period (*p* ≤ 0.001) and between the 6 h period and the 1 h period (*p* = 0.023). However, there was no significant difference between the 12 h period and 6 h period (*p* = 0.341; Table 4).

There was a statistically significant difference in the average MEWS based on weight classification. Underweight patients had a higher MEWS compared to the other weight groups. Underweight patients also had the worst outcomes with 100% mortality with no ROSC achieved following CPR. There was no statistical difference in the means of the MEWS at 12 h, 6 h, and 1 h periods prior to cardiac arrest event in the other subgroups which included sex, race, mortality outcomes, COVID-19 and non-COVID-19 patients.

## 4. Discussion

In this retrospective study, we were able to identify a significant rising trend in MEWS over a period of 12 h prior to a cardiac arrest event. We also found that underweight patients had higher average MEWS compared to other weight classes. In addition, patients with lower MEWS were more likely to survive cardiac arrest, although this was not statistically significant. There is a high incidence of in hospital cardiac arrest, with over 200,000 cases reported in the United States each year (1). Despite the possibility of identifying at-risk patients in a timely manner, patients are usually identified late. Our study suggests that a rising trend in MEWS may be a viable tool in identifying at-risk patients and preventing a cardiac arrest event.

We found that from admission to the cardiac arrest event, MEWS significantly increased from the 12 h and 6 h marks to the 1 h mark. There was no significant difference in the average MEWS between the 12 h and 6 h periods before arrest. This may suggest that the 6 h period, with an average MEWS of 4.65, may be the significant time to intervene. This supports the findings from an earlier study that suggested 6 h prior to the event was the most favorable period to identify at-risk patients [11]. In the same study, the median MEWS from 6 to 0 h before the event was 6 (3–9) points for cases and 1 (0–3) point for controls (*p* < 0.001). Based on our study, once this threshold of a MEWS of 5 is reached, it is likely the clinician has less than 6 h to avert a significant event.

While we did not have a control group in our study, a nested case–control study compared patients who sustained cardiac arrest in the wards with those that did not [12]. The authors noted that although the MEWS at the time of admission was similar, MEWS significantly increased from admission in patients that sustained a cardiac arrest compared to those that did not suffer a cardiac arrest [12]. This change can be identified as early as 48 h prior to the event [12]. These findings align with our results, which found that MEWS significantly increased between the 12 h/6 h periods and the one-hour period before cardiac arrest. Continuous monitoring of MEWS is necessary to identify any change that may occur.

Underweight patients had a higher average MEWS compared to other body BMI groups, but there was no statistical difference in other subgroups. This correlates with existing evidence indicating that being underweight increases the risk of cardiac arrest and adverse outcomes following cardiac arrest [13,14]. The other subgroups included race, sex, and presence of other comorbidities, such as hypertension, diabetes mellitus, COVID-19, or chronic kidney disease. This may indicate that the MEWS is a robust marker for clinical deterioration, and it is not affected by the patients’ underlying comorbidities. It is also possible that due to the small sample size, the study may be underpowered to detect statistical significance.

Patients who sustain an in-hospital cardiac arrest are unlikely to survive that admission even if ROSC is achieved following CPR [1,15], and our study aligns with these findings. Mortality in our patients remained high (79.3%). The low survival rate of this patient population emphasizes the urgent need for intervention systems like MEWS.

Implementing MEWS as an alert and tracking system has significantly reduced the incidence of cardiac arrest and emergent ICU admissions in other institutions [16,17]. In a study in Germany, the rate of cardiac arrests was significantly reduced from 5.3 to 2.1 per 1000 admissions, and there was a statistically significant decrease in unplanned ICU admissions by 0.6% [18]. Another study in the United Kingdom found that a MEWS-based track and trigger system significantly reduced the rate of in-hospital cardiac arrests by 9.4% compared to non-MEWS systems [19]. More recently, since the COVID-19 pandemic, telemedicine has garnered attention as a means of maximizing ICU and inpatient capacity with a focus on centralized remote monitoring techniques [20]. Implementing scoring systems such as the MEWS may have a significant role in the timely identification of patients at risk of cardiac arrest.

The method of deploying the application of MEWS is also important. Using automated and electronic systems appears more effective than paper forms. An electronic system was associated with a significant 9.8% reduction in events compared to paper [19]. Considering this information, incorporating the MEWS into an electronic medical record may be an effective method in the preventive strategy of in-hospital cardiac arrest. While our institute does not currently incorporate MEWS into patient monitoring, our data indicate that through monitoring a trend in MEWS, the identification of a rising trend may help a provider anticipate clinical deterioration culminating cardiac arrest. Timely intervention may have the potential to prevent cardiac arrest. A larger study would aid in assessing if patient outcomes and the incidence of in-hospital cardiac arrest may be reduced with the introduction of an alert system to the electronic medical systems already in use.

This study has had some limitations. Notably, this was a retrospective, single-center study based on chart review. The single center study may limit universality and external validity. The retrospective chart review may also introduce potential biases with data completeness. As described in the methods, unavailable data were assumed to be normal for some variables in order to avoid inappropriate overestimation of the MEWS.

Additionally, the small sample size of the study may limit the statistical power of the study and therefore the study may be underpowered to achieve statistically significant results when comparing certain values such as that observed in the trend between the 12 h and 6 h period MEWSs.

## 5. Conclusions

Our study showed a significant rising trend in MEWS over a 12 h period prior to a cardiac arrest event. Continuous monitoring of MEWS from admission could help predict and potentially prevent cardiac arrest. This retrospective chart review at a single institution supports the need for a larger scale study to better assess the role of MEWS in the prevention of in-hospital cardiac arrest.

## Figures and Tables

**Table 1:** The modified early warning score chart (MEWS) for single physiological parameters adapted from the Royal College of Physicians [8].

Physiological Parameter	Score						
	3	2	1	0	1	2	3
Respiratory rate	≤8		9–11	12–20		21–24	≥25
SpO_2_ Scale 1 (%)	≤91	92–93	94–95	≥96			
SpO_2_ Scale 1 (%) (History of LTOT)	≤83	84–85	86–87	88–92 ≥ 93 on air	93–94 on oxygen	95–96 on oxygen	≥97 on oxygen
Air or oxygen		Oxygen		Air			
Systolic Blood Pressure (mmHg)	≤90	91–100	101–110	111–219			≥220
Pulse (BPM)	≤40		41–50	51–90	91–100	111–130	≥131
Consciousness				Alert			CVPU
Temperature	≤35.0		35.1–36.0	36.1–38.0	38.1–39.0	≥39.1	

Note: BPM: beats per minute; CVPU: confusion, voice, pain, unresponsive; LTOT: long-term oxygen therapy; SpO_2_: saturation of peripheral oxygen.

**Table 2:** Demographic comparison by sex and race of age and average modified early warning scores (MEWS) at three different time points before cardiac arrest.

	*n* (%)	Average Age (Years)	*p*-Value	Average 12 h MEWS	*p*-Value	Average 6 h MEWS	*p*-Value	Average 1 h MEWS	*p*-Value
**Total cases**	87	69 ± 15		3.95 ± 2.4		4.65 ± 2.6		5.98 ± 3.5	
**Sex**									
Male	52 (59.8)	68.6 ± 15.2	0.62	3.9 ± 2.4	0.81	4.46 ± 2.6	0.42	5.67 ± 3.7	0.41
Female	35 (40.2)	70.3 ± 15.0		4.03 ± 2.5		4.46 ± 2.6		6.5 ± 3.2	
**Race**									
Black	17 (19.5)	61.5 ± 15.7	0.016	3.0 ± 2.6	0.09	4.31 ± 2.5	0.56	6.33 ± 4.2	0.70
White	70 (80.5)	71.2 ± 14.3		4.17 ± 2.3		4.73 ± 2.6		5.88 ± 3.4	

**Table 3:** Demographic comparison by body weight and comorbidities of average modified early warning scores (MEWS) at three different time points.

	*n* (%)	Average 12 h MEWS	*p*-Value	Average 6 h MEWS	*p*-Value	Average 1 h MEWS	*p*-Value
**Total cases**	87	3.95 ± 2.4		4.65 ± 2.6		5.98 ± 3.5	
**Weight class (BMI)**							
Underweight	7 (8)	5.33 ± 3.5	0.03	6.86 ± 2.1	0.066	10.4 ± 2.7	0.028
Healthy weight	33 (37.9)	3.1 ± 2.2		4.87 ± 3.1		5.33 ± 3.4	
Overweight	16 (18.4)	5.0 ± 2.3		4.0 ± 2.1		5.55 ± 2.7	
Obese	31 (35.6)	4.0 ± 2.2		4.19 ± 2.1		5.75 ± 3.8	
**COVID-19**							
Yes	15 (17.2)	4.08 ± 2.5	0.84	5.14 ± 4.1	0.44	6.50 ± 2.9	0.61
No	72 (82.8)	3.93 ± 2.4		4.55 ± 2.2		5.86 ± 3.7	
**Hypertension**							
Yes	47 (54)	3.8 ± 2.5	0.53	4.47 ± 2.5	0.5	5.81 ± 3.5	0.67
No	40 (46)	4.14 ± 2.3		4.86 ± 2.7		6.23 ± 3.7	
**Diabetes**							
Yes	24 (27.6)	4.32 ± 2.5	0.40	5.25 ± 2.5	0.18	6.33 ± 3.4	0.65
No	63 (72.4)	3.81 ± 2.4		4.39 ± 2.6		5.84 ± 3.6	
**CKD**							
Yes	9 (10.3)	3.0 ± 1.9	0.21	5.67 ± 1.9	0.21	4.33 ± 2.7	0.23
No	78 (89.7)	4.07 ± 2.4		4.52 ± 2.7		6.19 ± 3.6	
**Rhythm**							
Shockable	16 (18.4)	3.5 ± 1.9	0.22	3.93 ± 2.8	0.38	4.67 ± 3.1	0.56
Non-shockable	56 (64.4)	4.29 ± 2.4		4.94 ± 2.5		6.00 ± 3.2	
Undocumented	15 917.2)	3.15 ± 2.7		4.33 ± 2.7		6.64 ± 4.8	
**Outcome**							
Death during same admission	69 (79.3)	4.03 ± 2.4	0.56	4.84 ± 2.6	0.23	6.42 ± 3.5	0.11
Survived and discharged	18 (20.7)	3.65 ± 2.5		4.0 ± 2.5		4.62 ± 3.4	

Note: BMI: body mass index; CKD: chronic kidney disease.

**Table 4:** Pairwise comparisons of the average modified early warning score (MEWS) for each time period using the Bonferroni correction method.

(I) Time HrP	(J) Time HrP	Mean Difference in MEWS (I − J)	Standard Error	*p*-Value	95% CI of Mean Difference
**12**	6	−0.70	0.440	0.341	−1.76–0.36
	1	−2.03	0.494	<0.001	−3.22 to −0.84
**6**	12	0.70	0.440	0.341	−0.36–1.76
	1	−1.33	0.495	0.023	−2.53 to −0.14
**1**	12	2.03	0.494	<0.001	0.84–3.22
	6	1.33	0.495	0.023	0.14–2.53

CI: confidence interval; HrP: hour period.

## Data Availability

Datasets used for the study are available at the Allegheny Health Network’s Health System.
